# Investigating the Relationships between COVID-19 Quality of Life, Loneliness, Happiness, and Internet Addiction among K-12 Teachers and School Administrators—A Structural Equation Modeling Approach

**DOI:** 10.3390/ijerph19031052

**Published:** 2022-01-18

**Authors:** Turgut Karakose, Tuncay Yavuz Ozdemir, Stamatios Papadakis, Ramazan Yirci, Secil Eda Ozkayran, Hakan Polat

**Affiliations:** 1 Department of Educational Sciences, Faculty of Education, Kutahya Dumlupinar University, Kutahya 43100, Turkey; turgut.karakose@dpu.edu.tr; 2 Department of Educational Sciences, Faculty of Education, Firat University, Elazig 23119, Turkey; tyavuz23@gmail.com (T.Y.O.); hakanpolat@firat.edu.tr (H.P.); 3 Department of Education, University of Crete, 74100 Rethymno, Greece; 4 Department of Educational Sciences, Faculty of Education, Sutcuimam University, Kahramanmaras 46050, Turkey; yirci@ksu.edu.tr; 5 Department of Educational Sciences, Faculty of Education, Bartin University, Bartin 74100, Turkey; sekartal@bartin.edu.tr

**Keywords:** COVID-19, pandemic, COVID-19 quality of life, loneliness, Internet addiction, happiness, school administrator, teacher, K-12 education, structural equation modelling

## Abstract

It is well acknowledged that the roles of both school administrators and teachers have changed due to the global education crisis caused by COVID-19. During this challenging and critical period, it is essential to investigate how those working in the education sector who undertake strategic tasks for sustainable education are affected by the new conditions brought about by the COVID-19 pandemic. This study investigates the interrelationships between COVID-19 quality of life, loneliness, happiness, and Internet addiction. The research was designed according to the relational survey model, was conducted with 432 school administrators and teachers working in K-12 schools. The research data was collected through online questionnaires, and structural equation modelling (SEM) was used to test and analyze proposed hypotheses. The study’s results revealed a positive relationship between the COVID-19 related quality of life and loneliness, and that loneliness significantly positively predicts Internet addiction. In this context, due to the impact of COVID-19 on the life quality, the participants’ loneliness levels significantly increased, and this increase in loneliness caused them to become addicted to using the Internet. Interestingly, it was also determined that a positive relationship exists between loneliness and happiness and that as the loneliness of individuals increased, their level of happiness also increased. In many studies conducted prior to the COVID-19 pandemic, a negative relationship was revealed between loneliness and happiness. In the current study conducted during the pandemic, the relationship between the two variables was positive. SEM results revealed that COVID-19 directly affects the quality of life, Internet addiction, loneliness, and happiness of school administrators and teachers. Furthermore, it was determined that Internet addiction indirectly affects the relationship between loneliness and happiness.

## 1. Introduction

Humankind has faced many pandemics of different types from the past to the present, and significant numbers of people have lost their lives during these times. Successive plagues related to the Black Death (c. 1347–1351) saw humanity left practically helpless and powerless, as did the Spanish flu, which resulted in the death of millions of people worldwide [1]. These pandemics, which emerged suddenly and spread rapidly, brought radical societal changes. Today, humanity is again struggling with a deadly pandemic, COVID-19, and the psychosocial and economic effects of this pandemic have affected practically every aspect of modern-day human life, and as such, have been profoundly and widely felt on a global scale.

The COVID-19 disease, which first appeared in Wuhan in China’s Hubei province in December 2019, was soon declared a global pandemic by the World Health Organization (WHO) in March 2020. It led to an unprecedented worldwide public health crisis [2,3,4,5] in just a short time. Most governments reacted similarly, if not together, against the spread and effect of the virus, recognizing that if its spread were left uncontrolled, the ensuing loss of life would be devastating. As a result, degrees of quarantine were imposed worldwide via government mandate and local regulation, albeit with varying degrees of scope and strictness of application [6].

As a result, the adverse effects of the pandemic suppressed many areas of human social life, such as health, education, the economy, sports, and religious activities, which effectively impacted practically everyone’s lives and harmed the daily operations of most companies and institutions in some way. From this perspective, the COVID-19 global pandemic has impacted the social dimensions of life more than the individual. Therefore, it was perhaps inevitable that COVID-19 would soon become a field of significant research activity for many disciplines [7]. Since the beginning of the COVID-19 pandemic, it has been observed that human behaviours and habits, shaped from hundreds of years of experience, underwent a rapid and radical change in just a brief period. The situation that emerged due to these changes was the “new normal” [8]. People unprepared for the chaos caused by the pandemic were directly affected in terms of their health and economic and sociocultural aspects [4,9,10,11,12,13]. The global infectious diseases of such pandemics negatively affect the physical, social, and psychological functions of both individuals and societies as a whole and thereby result in a widespread impact of significant and essential consequences [13,14]. In addition, during such pandemics, it becomes essential to understand the reflections of people’s psychological states [15].

This study’s primary focus is to examine the interrelationships between COVID-19 quality of life, loneliness, happiness, and Internet addiction among school administrators and teachers. The spread of the epidemic, schools’ closure and the transition to distance education started a new process for students, teachers, and school administrators [16]. Uncertainty brought by the pandemic has caused some crises in education, and school administrators and teachers have been negatively affected by this crisis. Dealing with many problems such as the deterioration of work-life balance, psychological disorders, and the negative impact on the quality of life with COVID-19 has also affected teachers’ and school administrators’ happiness levels. When the academic databases are examined, it is seen that very few of them have a positive perspective on the effects of the pandemic. It also seems that there are a few studies in positive education during the pandemic [17]. Another problem is that scientific literature mainly focuses on clinical the effects of the COVID-19 rather than the psychosocial effects. It is undeniable that if the psychological aspect of the fight against the epidemic is neglected, the struggle will be incomplete.

Teachers and school administrators, who ensured the continuation of educational activities throughout the epidemic, have undertaken essential responsibilities. It has been considered essential to examine the relationships between the quality of life, happiness levels, loneliness, and Internet addiction levels of teachers and school administrators, who have continued their responsibilities such as being a source of motivation for students, leading and being a role model during the pandemic period [18]. When the COVID-19 literature is examined, it has been seen that there are very few studies focusing on the psychological state of school administrators and teachers during the epidemic. This specific research is the outcome of a modest effort to fill this gap in the field.

## 2. Literature Review

In 1948, the World Health Organization defined health as “not merely the absence of disease but a state of complete physical, mental and social well-being” [19]. It was also emphasized that people’s lives are meaningful and should be experienced at a certain level of “life quality”. The World Health Organization has conducted or supported many studies since the 1980s to measure and evaluate the quality of life [20]. The restrictions concerning the COVID-19 pandemic, to which people were previously largely unaccustomed, undoubtedly harmed their quality of life.

The concept of quality of life dates back to ancient times in sociology and medicine. Plato’s “Republic” (427–347 BC) and Aristotle’s “Nicomachus Ethics” (384–322 BC), both written in ancient times, include serious discussion on the quality of life and shows how the concept has long existed from our distant past right through until today [21]. The World Health Organization defines the quality of life as the standard of living that individuals perceive in line with their culture, values, goals, and expectations from life [22]. In addition, quality of life is seen as a comprehensive health outcome that reflects individuals’ perceptions of physical and mental health, social relationships, and general well-being [23,24]. In the related literature, it has been suggested that quality of life is an essential predictor of the permanence of overall health and well-being [19]. Despite the perceived importance of quality of life in health and medicine, there is an ongoing conceptual and methodological debate about its true meaning and what it should measure [25].

The quarantine, lockdown, and curfew practices introduced during the COVID-19 pandemic effectively cut off or dramatically curtailed most forms of direct contact between people not of the same household. This situation exacerbated feelings of loneliness considerably more during the pandemic [26,27,28]. Although loneliness is a universal problem, the relevant literature contains numerous studies that have been conducted on the topic of loneliness in recent years. In the foundation of this feeling, a more intense perception of loneliness exists connected to sociocultural structure and dynamics [29]. Sigmund Freud first used the term loneliness in 1939 to describe a person’s internal structure, which can change completely after the experience of loneliness. Years later, Sullivan detailed Freud’s definition of loneliness, arguing that humans are social animals that need contact and that loneliness is considered the result of this need going unmet [30]. Individuals can experience inadequate relationships and social lives that are not perceived as individually satisfactory can profoundly affect their lives. This situation has caused individuals to isolate themselves from society further and thereby experience profound loneliness [31,32].

Although loneliness is a known human emotion, it is also considered a complex situation experienced differently by each individual, and therefore cannot be explained with a single reason. Measures and practices that aim to eliminate the feelings of loneliness may differ according to the individual [33]. The definition of loneliness is a situation in which interpersonal relationships deteriorate, social participation decreases, and an individual can feel lonely and misunderstood [34]. The changing living conditions can exemplify this due to mandatory social restrictions introduced during the COVID-19 pandemic. During this process, individuals became isolated within closed areas, and the increasing amount of time they spent being inactive also directly affected their quality of life [35]. Among the measures implemented during the pandemic, the most common was social isolation, a measure that dramatically changed the lifestyle and habits of many individuals worldwide [36]. As a result, social relations decreased, and loneliness increased due to this enforced period of extended isolation [37]. Studies have shown that high-level restrictions can result in loneliness due to quarantine measures and that loneliness is associated with higher psychosocial distress and lower life satisfaction [38].

Another behavioural change observed in people during the COVID-19 pandemic was digital media usage. With people primarily forced to stay in their homes during the pandemic, many started to spend more time using the Internet [39,40,41]; however, in utilizing the Internet, many borders and barriers between people were effectively removed, or at least circumnavigated. In addition, with the speed of today’s Internet-based communication, the medium has helped make people’s lives easier and, as such, has become a tool associated with an increased quality of life. With its various functionality and ubiquitous spread, digital media has become a phenomenon that needs to be considered sociologically, psychologically, and socially [42].

The 2020 World Internet Usage and Social Media Statistics report published by We Are Social stated that the number of people using the Internet worldwide had increased by 7% (298 million new users) compared to data reported in only January 2019, reaching a new total of 4.54 billion global users. The average Internet user spends 6 h and 43 min online every day, which is 3 min less than in the previous year but equal more than 100 days of connected time per Internet user per year. Considering that an average person has about 8 h of sleep every 24, their Internet usage now constitutes more than 40% of each person’s waking time [43].

In the related literature, Internet addiction has been defined as an impulse control disorder unrelated to using intoxicating substances [44]. It has been stated that people addicted to using the Internet cannot control their usage and experience feelings of anxiety when they are unable to do so. In addition, it is known that even if they make efforts to reduce their Internet usage, they may fail in that endeavour and subsequently try to hide their continued usage [45]. It became inevitable that irregular use of the Internet, at more moderate and controlled levels, has fast become a thing of the past, and that has been especially notable during the COVID-19 pandemic period [46]. However, during this period, people have been essentially forced to spend more time at home and conduct most of their work remotely using online tools, and a need for increased social usage has significantly increased many people’s daily Internet usage. The researches on the relationship between internet addiction, happiness and loneliness have different results in different samples. For instance, Alqahtani et al. [47] found that there is no relationship between internet addiction, loneliness and life satisfaction (*p* > 0.01) in the Saudi population; however, Erol and Cirak [48] found a significant correlation between loneliness and Internet addiction in the Turkish context. Ansari et al. [49] found that participants’ happiness scores were statistically significantly associated with internet addiction.

Various research has demonstrated that the COVID-19 pandemic has deeply affected people’s moods and that this effect has been primarily negative [50,51,52]. The negative emotional states experienced by people during the pandemic have also reduced their happiness levels [53,54,55]. One of the most basic human needs is happiness, as when individuals are happy, they feel more prosperous and more secure. Happiness has the potential to make every moment of life different [56]. In essence, an individual experiences more positive emotions than negative, and the more satisfied they are with their life in general, the greater the level of happiness they will likely experience. However, the negative impact of living with a contagious and pandemic disease on happiness is undeniable [57,58]. Therefore, it has been stated that experiencing fear of COVID-19 can directly reduce happiness amongst individuals and negatively affect their mental health [59]. Curfews and lockdowns imposed at the national and/or local level, especially in Europe and the United States, have led to significant increases in searches on topics such as boredom, loneliness, anxiety, and sadness in the Google search engine [60].

During the COVID-19 pandemic, people in many countries worldwide experienced some form of negative emotional experience. The concept of “new normal” that emerged with COVID-19 has dramatically affected the individuals’ life quality, and therefore their perception of loneliness. At the same time, with the temporary (and some permanent) closure of businesses and services experienced during the pandemic period, an inevitable rise in Internet addiction amongst individuals has been seen to increase. As such, their happiness expectations have also significantly changed.

## 3. Materials and Methods 

### 3.1. Purpose of the Study and Hypotheses

The current study examined the interrelationships between COVID-19 quality of life, loneliness, happiness, and Internet addiction among school administrators and teachers. The Structural Equation Model (SEM) was selected, a comprehensive method that discovers and confirms causality between various variables and combines different analyses such as multiple regression, path analysis, and factor analysis [61]. In this context, the hypotheses developed in line with the general purpose of the current research are as follows:

**Hypothesis** **1** **(H1):** *COVID-19′s impact on quality of life is positively associated with loneliness*.

**Hypothesis** **2** **(H2):** *Loneliness is positively related to Internet addiction*.

**Hypothesis** **3** **(H3):** *Loneliness is negatively related to happiness*.

**Hypothesis** **4** **(H4):** *Internet addiction is negatively related to happiness*.

**Hypothesis** **5** **(H5):** *Internet addiction indirectly affects the relationship between loneliness and happiness*.

**Figure 1:**
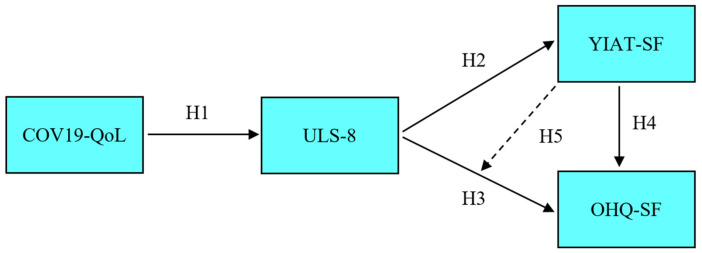
Hypothesized relationships of the research model [COV19-QoL = COVID-19 Quality of Life; ULS-8 = Loneliness; YIAT-SF = Internet Addiction; OHQ-SF = Happiness].

Figure 1 presents a hypothetical model for the relationships between the variables examined in the study.

### 3.2. Study Design

The research is a relational study that examines the COVID-19 related quality of life and the relationships between loneliness, happiness, and Internet addiction levels amongst teachers and school administrators. A relational study was selected to examine relationships between variables [62,63]. Within the scope of the current research, a multifactorial predictive correlational design was employed. This design analyzed direct and indirect causal relationships between independent and dependent variables [61,64]. In this context, the study examines in detail the effect of COVID-19 as experienced by school administrators and teachers on their quality of life and examines the direct and indirect relationships between loneliness, happiness, and Internet addiction.

### 3.3. Participants

This study, conducted using Structural Equation Modeling (SEM), was deemed appropriate to determine the sample size required by structural modelling, as SEM tests are sensitive to sample size [65]. In this context, it has been stated that it is appropriate for the sample size to be ten times the number of items (variables) [66]. Accordingly, a sample size of at least 320 participants was deemed sufficient for the current study, considering its total of 32 variables (items). It was also considered possible that some participants may complete the data collection instrument incompletely or incorrectly, and therefore the scale was applied to a more significant number of participants to reach a sufficient sample of acceptable data.

Using simple random sampling, 454 people were determined and included. According to the Ministry of Education statistics, 7.452 teachers work at 581 Kutahya province (Turkey) schools. The researchers defined the 88 schools randomly and contacted the principals via email and telephone. The research purpose was explained, and they were kindly requested to participate in the research. The scales were submitted electronically via Google Forms. As a result of the initial data analysis, 22 were excluded from the dataset due to extreme values. Therefore, the research was conducted with 432 school administrators and teachers working in K-12 schools during the 2021-2022 academic year in Kutahya. The sociodemographic characteristics of the participants are presented in Table 1.

When the summary data in Table 1 are examined, it can be seen that the ratio of male and female participants is close to each other. Additionally, the rate of teachers and administrators infected with COVID-19 was low (7.9%). This situation may be explained by the high “vaccination” rates, also shown in Table 1. According to data from the Turkish Ministry of National Education, the rate of teachers having received two doses of a COVID-19 vaccine has reached 86%. According to the national data, as of 8 November 2021, the entire Turkish population’s vaccination rate was 79.25% [67]. Therefore, the rate of teachers having received a double vaccine dose is higher than that of the general population. Another interesting aspect of the data shown in Table 1 is that the rate of participants whose daily Internet usage was 5 h or more was 10.2%. 

### 3.4. Data Collection and Data Analysis

#### 3.4.1. Measurements

Within the scope of the research, the study’s data were collected using the COVID-19 Quality of Life Scale (COV19-QoL), the Oxford Happiness Questionnaire Short Form (OHQ-SF), the Short-form UCLA Loneliness Scale (ULS-8), the Short form of Young’s Internet Addiction Test (YIAT-SF), as well as a Personal Information Form developed by the researchers for the study.

The scales used in the study were reviewed and their application approved by the Kutahya Governorship and Provincial Directorate of National Education, with legal permission granted for the study to be performed with K-12 teachers and school administrators (Permit: 53490996-44-E.7646169).

##### COVID-19 Quality of Life Scale (COV19-QoL)

The original COV19-QoL scale was developed by Repišti et al. [68], and Sümen and Adibelli [69] subsequently conducted a Turkish validity and reliability study. The Turkish version (COV19-QoLTR) consists of six items covering mental health’s leading quality of life areas. The scale is a five-point, Likert type (1 = *Strongly disagree* to 5 = *Strongly agree*) instrument that aims to evaluate the feelings and thoughts of individuals during the past seven days. There are no reverse-coded items on the scale. The scale’s score is calculated by dividing the total score by the number of items. A higher score indicates a more significant impact of the pandemic on the individual’s quality of life. The Cronbach’s alpha coefficient of the COV19-QoLTR scale was 0.910 for the data obtained from the general population [69].

##### Oxford Happiness Questionnaire Short Form (OHQ-SF)

The OHQ-SF scale, which was developed initially by Hills and Argyle [70] to measure an individual’s level of happiness, was adapted to the Turkish context by Dogan and Cotok [71]. The scale consists of seven items formed as a five-point, Likert-type instrument, and both Item 1 and Item 7 are reverse-coded. High scores achieved from the scale indicate a high level of happiness. The internal consistency coefficient of the one-dimensional scale was calculated as 0.74, and the test-retest reliability coefficient was 0.85.

##### Short-Form UCLA Loneliness Scale (ULS-8)

The ULS-8 scale was originally developed by Hays and DiMatteo [72] and later adapted to the Turkish context by Yıldız and Duy [73]. The scale consists of seven items as a five-point, Likert-type instrument scale, with Item 5 being reverse-coded. As a result of Exploratory Factor Analysis, the scale consisted of a single dimension. Confirmatory Factor Analysis revealed that the unidimensional structure showed a good fit (χ^2^ = 27.12, *SD* = 14, χ^2^/*SD* = 1.94, RMSEA = 0.06, RMR = 0.03, SRMR = 0.04, GFI = 0.97, AGFI = 0.95, CFI = 0.98), NFI = 0.96, NNFI = 0.97). A low score from the ULS-8 scale indicates a low feeling of loneliness, while a high score indicates increased loneliness. The internal consistency coefficient of the scale was calculated as 0.74, with a test-retest reliability coefficient of 0.84.

##### Short-Form of Young’s Internet Addiction Test (YIAT-SF)

The YIAT-SF scale was adapted to Turkish by Kutlu et al. [74]. The scale is constructed as a five-point, Likert type (1 = *Never* to 5 = *Always*) instrument with 12 items formed within a single dimension. There are no reverse-coded items in the YIAT-SF scale. The combined score obtainable from the scale can vary between the lowest score of 12 and the highest of 60, with a higher score indicating a higher level of Internet addiction. The Cronbach’s alpha coefficient of the scale was calculated as 0.91 for university students and 0.86 for adolescents.

#### 3.4.2. Data Analysis

After obtaining the necessary permissions to conduct the research, the data collection instruments were sent to the participants online. The obtained data were then analyzed using IBM’s SPSS package program, with the Maximum Likelihood method applied using the SPSS Amos module. Sample size, multicollinearity problem, normality, and extreme values were also examined as prerequisites for applying the Structural Equation Model (SEM) [75]. First, the Z-score values of the variables were excluded from the dataset, with 22 data outliers found not between −1 and +1. Next, correlation analysis was performed to determine multicollinearity problems among the variables. Where correlation values are below 0.90, it may be said that no multicollinearity problem exists [75]. In the analysis performed in the current study, correlations between the variables were examined, and it was determined that the correlation coefficients between the variables were all below the 0.90 value (see Table 2). In line with this data finding, it was determined that no multicollinearity problem was found to exist between the variables. To determine the multicollinearity problem, the VIF and tolerance values of the independent variables were examined, and it was determined that the values did not cause any multicollinearity problems (see Table 3). Another prerequisite for SEM analysis is that the data exhibits normal distribution. The analysis examined the kurtosis and skewness values of the variables, and it was determined that the dataset was normally distributed (see Table 4).

Therefore, it was taken into account that the data obtained within the scope of the research showed normal distribution, that the sample size was sufficient, and that neither linearity nor multicollinearity problems were present. The covariance matrix and the Maximum Likelihood methods were applied to test the measurement and structural models. In analyzing the data, the impact of COVID-19 on quality of life and the measurement models of the variables of happiness, loneliness, and Internet addiction were tested with Confirmatory Factor Analysis (CFA). Whether or not the measurement models were validated was examined using the Chi-square (χ^2^)/*SD*, GFI, AGFI, CFI, RMSEA, IFI, and TLI (NNFI) fit indices. Finally, whether or not the proposed hypothetical model was confirmed was then examined according to the specified fit indices. Diagrams were created using Flowchart Maker & Online Diagram Software [76].

## 4. Results

The findings obtained from the correlation analysis of the data collection tools applied in the study are presented in Table 2.

Table 2 shows that the COVID-19 related quality of life had a positive relationship with happiness (*r* = 0.160, *p* < 0.01), whereas loneliness had a negative relationship with COVID-19 related quality of life (*r* = −0.221, *p* < 0.01), and Internet addiction had a significant negative relationship with COVID-19 related quality of life (*r* = −0.096, *p* < 0.01). In addition, negative relationships were revealed between happiness and loneliness (*r* = −0.279, *p* < 0.01), and between happiness and Internet addiction (*r* = −0.094, *p* < 0.01). Finally, the relationship between loneliness and Internet addiction was positive (*r* = 0.0149, *p* < 0.01) at a significant level. When these findings were evaluated, the correlations between the variables ranged from −0.279 and 0.149. These values also show that no multicollinearity problem existed between the study variables.

Table 3 presents the VIF and tolerance values for the independent variables in the measurement model.

Cokluk et al. [75] emphasized that VIF values should be less than ten and tolerance values greater than 0.10. Accordingly, when Table 3 is examined, it can be seen that no multicollinearity or multicollinearity problems were found between the variables examined in the current study.

In Table 4, the mean, standard deviation, skewness, and kurtosis values of the measurement model variables.

Finally, the data were examined in order to determine whether or not it was normally distributed. According to Table 4, the skewness value was 0.933, and the kurtosis value was 0.867 for the Impact of COVID-19 on Quality of Life. For the Oxford Happiness Scale Short Form, the skewness value was 0.520, and the kurtosis value was −0.628. For the UCLA Loneliness Scale Short Form, the skewness value was −0.124, and the kurtosis value was −0.549. For the Short form of Young’s Internet Addiction Scale, the skewness value was 0.051, and the kurtosis value was −0.863. In light of these results, it may be said that the skewness coefficients of the variables were found to be within acceptable limits and that the data exhibited a normal distribution.

When Table 4 is examined, the scores obtained from the COVID-19 Quality of Life Scale on the impact of the pandemic on the quality of life can be seen to vary between 1 and 4. The participants’ opinions presented an average score of X = 1.1539 (*SD* = 0.7645), which may be interpreted as the pandemic having impacted little on the participants’ quality of life. The scores obtained from the Oxford Happiness Questionnaire Short-Form scale ranged from 1 to 5, with an average value of X = 2.4074 (*SD* = 1.132), which points to a moderate happiness score during the COVID-19 pandemic. Although the scores obtained from the Short-Form UCLA Loneliness Scale varied between 1 and 5, the participant opinions averaged at X = 3.2963 (*SD* = 0.9849), which may be interpreted as a high level of loneliness experienced the pandemic. The scores obtained from the short form of Young’s Internet Addiction Test ranged from 1 to 5, and the participants’ opinions had an arithmetic mean of 3.213 (*SD* = 1.119), which are considered above the medium level. As known, the Internet addiction of individuals has increased during the COVID-19 pandemic. In addition, when the kurtosis and skewness values of the dataset were examined, it was determined that the data exhibited a normal distribution.

### 4.1. Assessment of Measurement Model

Before assessing the structural model, Cronbach’s Alpha values of the scales were examined for construct reliability. The reliability values (Cronbach’s Alpha) were 0.84, 0.89, 0.77 and 0.89 as presented by Table 6. Therefore the reliability of the scales were confirmed. In addition, we examined the validity of the scales used in the research, and confirmatory factor analysis was conducted. Confirmatory Factor Analysis of measurement tools used in SEM research should be repeated with the existing dataset. For this purpose, relevant analyses of the scales applied in the study were conducted. Τhe fit indices of this analysis are presented in Table 5.

The COVID-19 Quality of Life Scale (COV19-QoL) is a one-dimensional scale of six items. The measurement model was tested with first-level CFA, and it was found that all paths related to the six items in the scale were statistically significant at the 0.01 level. According to the CFA results, the *t*-values of the scale varied between 10,041 and 13,702. Values for the COV19-QoL scale were established as χ^2^/*SD* = 3.292, GFI = 0.982, AGFI = 0.947, IFI = 0.990, TLI = 0.977, CFI = 0.989, and RMSEA = 0.073 (see Table 5). According to the CFA results, it was determined that the GFI, IFI, TLI, CFI, and RMSEA values had a good fit and that the χ^2^/*SD* value was revealed as being in the acceptable range.

The Oxford Happiness Questionnaire Short Form (OHQ-SF) is a one-dimensional scale consisting of seven items. With CFA, all of the paths except for Item 7 in the scale were statistically significant at the 0.01 level. Since the path for Item 7 was not statistically significant, the item was excluded from the analysis. In the subsequent analysis, it was determined that the *t*-values of the scale varied between 10,041 and 13,702. In addition, the OHQ-SF scale was found to have values of χ^2^/*SD* = 1.923, GFI = 0.987, AGFI = 0.969, IFI = 0.984, TLI = 0.972, CFI = 0.983, and RMSEA = 0.046 (see Table 5), and that all of these values can be said to have a good fit.

The Short-Form UCLA Loneliness Scale (ULS-8) consists of one dimension and seven items. With CFA, it was seen that all paths related to the seven items in the scale were statistically significant at the 0.01 level. According to the CFA results, the *t*-values of the scale varied between 10,041 and 13,702. The values for the ULS-8 scale were χ^2^/*SD* = 2.932, GFI = 0.979, AGFI = 0.951, IFI = 0.975, TLI = 0.956, CFI = 0.975, and RMSEA = 0.067 (see Table 5), and all of these values can be said to have a good fit.

The Short-Form of Young’s Internet Addiction Test (YIAT-SF) is a one-dimensional scale consisting of 12 items. The measurement model was tested with CFA, and all paths related to the 12 items in the scale were found to be statistically significant at the 0.01 level. According to the CFA results, the *t*-values varied between 10,041 and 13,702. The values of the YIAT-SF scale were established as being χ^2^/*SD* = 3.974, GFI = 0.928, AGFI = 0.896, IFI = 0.942, TLI = 0.924, CFI = 0.941, and RMSEA = 0.083 (see Table 5). According to the CFA results, it was determined that the χ^2^/*SD*, GFI, IFI, TLI, and CFI values were within the acceptable range and that the RMSEA value had a good fit.

Construct validity was also examined using average variance extracted (AVE) and composite reliability (CR) in the research. Composite reliability (CR) score should exceed the acceptable value of 0.7 for all factors, and the average variance extracted (AVE) must be greater than 0.5 for all variables [77,78,79]. AVE and CR scores can be seen in Table 6.

It can be seen in Table 6 that Cronbach’s Alpha scores range from 0.77 to 0.89. AVE scores are above 0.50, and CR scores are above 0.70 for each construct, showing the constructs’ consistency. In the light of these results, we can state that convergent validity was established.

### 4.2. Assessment of the Structural Model

For research purposes, the direct relationship of COVID-19′s effect on the quality of life with loneliness and loneliness with happiness and Internet addiction were investigated according to SEM (see the hypothetical model presented in Figure 1). In addition, the mediating effect of happiness in the relationship between loneliness and Internet addiction was also examined. Testing the final model revealed that the *t*-values ranged from −14,619 to 24,824. In addition, the standardized path coefficient between the COVID-19 related quality of life and loneliness in the model was found to be 0.45, and the standardized path coefficient between loneliness and happiness was found to be 0.01. The fit indices of the hypothetical model can be seen in Table 7.

**Figure 2:**
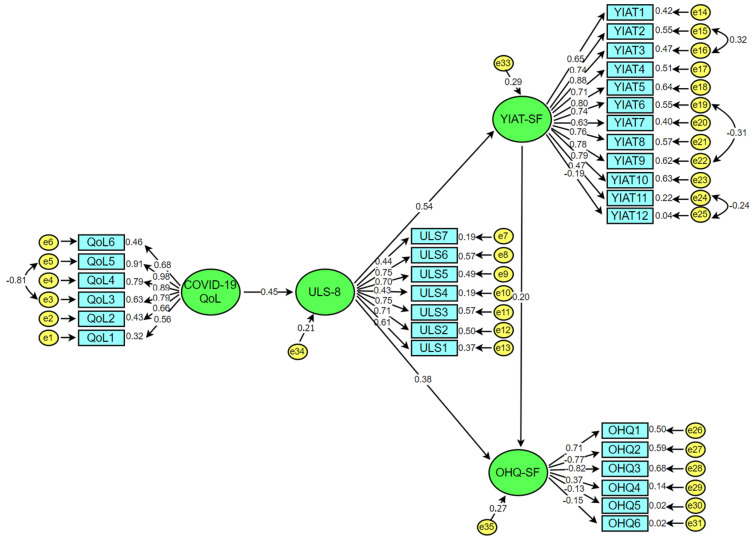
Final hypothesized model.

The fit indices of the hypothetical model were calculated as χ^2^/*SD* = 3.66. In addition, the values were determined as GFI = 0.89, AGFI = 0.89, IFI = 0.90, TLI = 0.89, CFI = 0.89, and RMSEA = 0.08. When the fit indices of the model were examined, it can be seen that all values had an acceptable fit except GFI and TLI values, however; the GFI and TLI values in the model are very close to the acceptable fit. Schermelleh-Engel & Moosbrugger [85] state that it is always possible that a model may fit the data, although one or more fit measures may suggest a bad fit. As a result, the analyses showed that the final hypothetical model outlined in the research could be said to be confirmed. In this context, the proposed SEM is presented in Figure 2.

The standardized regression coefficient between the COVID-19 related quality of life and loneliness was calculated as 0.45. This value shows a positive relationship between the COVID-19 related quality of life and loneliness, which explained 47.6% of the variance of loneliness. Kline [65] stated that an effect size of around 0.10 is considered “small”, whereas an effect size of around 0.30 is “medium”, and an effect size of around 0.50 is described as “large”. Accordingly, the standardized regression coefficient between the COVID-19 related quality of life and loneliness can have a medium effect size. This result shows that the COVID-19 related quality of life positively and significantly predicts loneliness. In other words, as the impact of COVID-19 on the quality of life of individuals increases, their level of loneliness also increases to a significant degree. This result revealed that the first hypothesis of the research (H1: “COVID-19′s impact on quality of life is positively associated with loneliness”) is confirmed.

The standardized regression coefficient between loneliness and Internet addiction was 0.54. This value shows that a positive relationship exists between loneliness and Internet addiction. Loneliness was shown to explain 20.3% of the variance of Internet addiction. The standardized regression coefficient between loneliness and Internet addiction shows a large effect size, and it can therefore be said that loneliness predicts Internet addiction positively and with statistical significance. This result reveals that the second hypothesis of the research (H2: “Loneliness is positively related to Internet addiction”) is confirmed.

In Figure 2, it can be seen that the standardized regression coefficient between loneliness and happiness is 0.38. This finding reveals a positive and moderate relationship between loneliness and happiness. In other words, as the loneliness of individuals increased, the level of their happiness also increased. Loneliness was shown to explain a 36.2% variance of happiness, and therefore that it has a moderate effect on the happiness levels of individuals. This result revealed that the third hypothesis of the research (H3: “Loneliness is negatively related to happiness”) is rejected. While in many studies prior to the pandemic, a negative relationship was established between loneliness and happiness, in the current study conducted during the COVID-19 pandemic, the relationship between the two variables was positive. This may be explained by those individuals with increased levels of loneliness, being in isolation from their normal social life, having felt more positive regarding the effect of their loneliness, having considered that they were more protected and safer from the negative effects of the virus. In addition, the satisfaction of individuals who had the opportunity to spend more time with their families during the pandemic may have affected this result. Therefore, the positive relationship between happiness and loneliness can also be remarkable in revealing an indirect effect of the pandemic on the mood of individuals.

When Figure 2 is examined, it can be seen that the standardized regression coefficient between Internet addiction and happiness was 0.20. This finding reveals a positive relationship between Internet addiction and happiness, and Internet addiction explained a 36% variance in happiness. In light of this finding, it may be said that Internet addiction positively and significantly predicts the happiness of individuals to a statistical degree. This result reveals that the fourth hypothesis of the research (H4: “Internet addiction is negatively related to happiness”) is rejected.

The increased Internet usage levels of individuals during the COVID-19 pandemic period can be interpreted as having protected individuals from some of the negative effects of the pandemic and thereby contributed positively to their happiness levels. This result reveals that the fifth hypothesis of the research (H5: “Internet addiction indirectly affects the relationship between loneliness and happiness”) is confirmed. In this context, Figure 2 shows that Internet addiction (0.54 * 0.20 = 0.108) had a 10.8% indirect effect on the relationship between loneliness and happiness. In the final hypothetical model tested through SEM, it was found that the COVID-19 related quality of life predicted the loneliness level of individuals, and as the loneliness level of individuals increased, their Internet addiction and happiness levels also increased.

## 5. Discussion

The research findings revealed a strong positive relationship between the impact of COVID-19 on the quality of life and the loneliness level of the participants. In this context, the individuals experienced increased loneliness as the impact of COVID-19 on their quality of life increased. Due to the rapid transmission and increased mortality from COVID-19, governments worldwide soon implemented extraordinary measures in an attempt to contain the next pandemic [86,87,88]. The quality of life was adversely affected due to curfews, travel restrictions, and isolation measures introduced during this period [89,90]. The impact of COVID-19 on the quality of life and the increasing sense of loneliness introduced new problems and exacerbated others [91,92,93]. For example, the current study investigated the impact of the COVID-19 pandemic on the quality of life of school administrators and teachers, and it was psychological effects that were primarily identified.

Furthermore, it was concluded that the school administrators and teachers experienced a negative psychological effect during the pandemic period. Experiencing negative situations such as difficulty in establishing healthy communication, emotional change, difficulty in anger control, and the desire to be alone is considered an adverse effect of COVID-19 on the quality of life [94]. This current study presents similarities with research conducted in several countries, with different samples and age groups [95,96,97].

The current research results revealed that the loneliness level of the participants during the COVID-19 pandemic predicted their Internet addiction positively and significantly. The statistical data obtained showed that the effect size between these two variables was large. These findings also coincide with other studies in the literature that examined the relationship between two variables. Alheneidi et al. [98] emphasized that a strong relationship existed between loneliness and problematic Internet usage and that feelings of loneliness tend to increase as the time spent using the Internet increases. In a study conducted by Deutrom, Katos, and Ali [99], it was revealed that loneliness could positively predict problematic Internet use. In the pre-COVID-19 period, similar findings were reached in studies focusing on the relationship between loneliness and Internet usage. For example, Kim et al. [100] and Karakose et al. [101] determined that feelings of loneliness caused increased Internet usage, and those individuals spent more time using the Internet to compensate for the socialization lacking from their real life. However, this situation can cause individuals to move away from healthier social activities and real-life [102,103]. During the COVID-19 pandemic, many people spent significantly more time using the Internet due to quarantine measures and curfews. While students and teachers had to conduct lessons over the Internet with online tools [103], businesses of all kinds utilized tools such as Zoom to conduct meetings more frequently than before and for extended periods [99]. Therefore, individuals’ increased Internet usage should not be perceived as a particularly negative or abnormal situation given the circumstances. Kraut and Burke [104] stated that positive or negative Internet usage relates to how individuals use the Internet, what they talk about with others when using the Internet, and whom they talk to. Hacker et al. [105] stated that during the COVID-19 pandemic, web-conferencing systems that utilize the Internet’s infrastructure made people’s everyday life easier, allowed meetings to occur that would otherwise not have happened, facilitated access to various daily activities, and provided a form of virtual “togetherness”. In the studies conducted in this area, it has been frequently emphasized that the opportunities presented during the pandemic period of compulsory digitalization and the Internet’s usage can also be said to have added value.

The results from the current research revealed a positive and moderate relationship between loneliness and happiness. Accordingly, it may be understood that the level of loneliness moderately affects the happiness levels of individuals. This result can be explained by individuals who spent more time with their families during the COVID 19 pandemic were satisfied overall with their situation. Working mothers, especially during the pandemic restrictions, had the opportunity to spend more time with their children. In this context, individuals who remained distant from the intensity of their everyday working life for a particular time may have been satisfied with the increased opportunity to spend time alone with their families. In a study on happiness, Petrovič et al. [106] compared happiness levels before and during the COVID 19 pandemic.

Contrary to expectations, at the end of their study, no significant change was detected in the participants’ happiness levels. Individuals who stayed at home and experienced loneliness during the pandemic period may have turned this complicated process into an opportunity by organizing different activities within the confines of their own homes. However, some studies in the literature concluded that the happiness levels of individuals, especially in older people, were found to have been negatively affected during the COVID-19 period and that decreased levels of happiness triggered anxiety, tension, fatigue, and stress can cause depression [107,108,109]. The psychological effects caused by COVID-19 have been shown to cause different moods in different age groups and this has forced policymakers and administrators to take necessary measures regarding different age groups.

Finally, the current study’s findings revealed a positive relationship between Internet addiction and happiness. Ballarotto et al. [110] determined that Internet usage was more frequently for entertainment, business, and educational activity during the COVID-19 pandemic. However, some studies conducted prior to the pandemic reported negative relationships between Internet addiction and happiness [111,112,113,114,115]. During the COVID-19 pandemic, Internet usage was shown to have increased dramatically across almost all age groups [116]. It may be stated that individuals use the Internet for shopping, attending online classes, managing jobs remotely and keeping in touch with people through online meetings, obtaining information or entertainment. This emerging situation is also interesting as it shows that Internet addiction has a multidimensional effect on the mood of individuals in both the pre-and post-COVID-19 pandemic.

### Limitations and Directions for Future Research

Although this study provides significant findings regarding the relationships between COVID-19 quality of life, loneliness, happiness, and Internet addiction, it undoubtedly has certain limitations. The research was conducted with a sample consisting only of school administrators and teachers, whilst it is known that emotional states associated with COVID-19 may vary among individuals working in different occupational groups. In addition, it may be said that the age group in the study sample was not homogeneously distributed and that the density consisted of participants between the ages of 21 and 40 years old (63.9%). However, the effects of the COVID-19 pandemic on individuals may differ in young, middle-aged, and older age groups. For this reason, conducting further detailed studies on the effects of the COVID-19 pandemic on larger samples from different age and occupational groups may present a more comprehensive evaluation of the subject. Another limitation of the current study is the period in which the data were collected. This experimental study was based on participants’ self-reports after all quarantine practices and restrictions had been lifted. In addition, when the data were collected, the number of COVID-19 cases in Turkey was low and the significance of the pandemic as a psychological threat was felt less than previously. This situation may have caused the participants to have presented a false image of their recollections. Therefore, future studies could examine the relationships between different variables in more detail using a longitudinal research approach.

## 6. Conclusions

In conclusion, according to Structural Equation Modelling, the current research proposed a new model from examining the relationships between the impact of COVID-19 on the quality of life, happiness, loneliness, and Internet addiction levels of teachers and school administrators. The variables observed in the proposed conceptual model were analyzed through path analysis, and the relevant model was then tested. The findings revealed that the impact of COVID-19 on the quality of life has a direct or indirect effect on the loneliness, happiness, and Internet addiction levels of the participants. As a result, it was seen that the fit values of the hypothesis model proposed within the scope of the research were at an acceptable level and that the model was confirmed.

## Figures and Tables

**Table 1:** Sociodemographic profile of the respondents.

Variables	Description	*f* (*N* = 432)	(%)
Gender	Male	182	42.1
	Female	250	57.9
Age (years)	20–30	36	8.3
	31–40	276	63.9
	41+	120	27.8
Occupation	Teacher	210	48.6
	Vice-principal	134	31.0
	Principal	88	20.4
Seniority (service years)	0–5	20	4.6
	6–10	106	24.5
	11–15	136	31.5
	16–20	102	23.6
	21+	68	15.7
COVID-19 infected (self)	Yes	104	24.1
	No	328	75.9
COVID-19 vaccinated (self)	Yes	398	92.1
	No	34	7.9
Daily Internet usage (hours)	1–2	56	13.0
	2–3	150	34.7
	3–4	118	27.3
	4–5	64	14.8
	5+	44	10.2

**Table 2:** Correlation values between scales.

Scale	COV19-QoL	OHQ-SF	ULS-8	YIAT-SF
COVID-19 Quality of Life Scale (COV19-QoL)	1	0.160	−0.221	−0.096
Oxford Happiness Questionnaire Short Form (OHQ-SF)		1	−0.279	−0.094
Short-Form UCLA Loneliness Scale (ULS-8)			1	0.149
Short form of Young’s Internet Addiction Test (YIAT-SF)				1

**Table 3:** VIF and Tolerance values of independent variables.

Scale	VIF	Tolerance
COVID-19 Quality of Life Scale (COV19-QoL)	1.033	0.968
Oxford Happiness Questionnaire Short Form (OHQ-SF)	1.033	0.968
Short-Form UCLA Loneliness Scale (ULS-8)	1.016	0.984

**Table 4:** Mean, standard deviation, skewness, and kurtosis values of the scales (*N* = 432).

Scale	Min	Max	X	*SD*	Skewness	Kurtosis
COV19-QoL	1.00	4.00	1.5139	0.7645	0.933	0.867
OHQ-SF	1.00	5.00	2.4074	1.132	0.520	−0.628
ULS-8	1.00	5.00	3.2963	0.9849	−0.124	−0.549
YIAT-SF	1.00	5.00	3.2130	1.119	0.051	−0.863

**Table 5:** Confirmatory Factor Analysis results of the scales.

Scale	χ^2^/*SD*	GFI	AGFI	IFI	TLI	CFI	RMSEA
COV19-QoL	3.292	0.982	0.947	0.990	0.977	0.989	0.073
OHQ-SF	1.923	0.987	0.969	0.984	0.972	0.983	0.046
ULS-8	2.932	0.979	0.951	0.975	0.956	0.975	0.067
YIAT-SF	3.974	0.928	0.896	0.942	0.924	0.941	0.083

**Table 6:** Reliability and convergent validity results.

Constructs	Cronbach’s Alpha	CR	AVE
COV19-QoL	0.84	0.72	0.68
ULS-8	0.77	0.71	0.67
OHQ-SF	0.89	0.70	0.66
YIAT-SF	0.89	0.71	0.66

**Table 7:** Fit indices of the structural model.

Fit Indices	Good Fit	Acceptable Fit	Structural Model
X^2^/sd	0 ≤ χ^2^/sd ≤ 3	3 ≤ χ^2^/sd ≤ 5	3.66
CFI	0.95 ≤ CFI ≤ 1.00	0.90 ≤ CFI ≤ 0.95	0.89
AGFI	0.90 ≤ AGFI ≤ 1.00	0.85 ≤ AGFI ≤ 0.90	0.89
GFI	0.95 ≤ GFI ≤ 1.00	0.90 ≤ GFI ≤ 95	0.89
TLI	0.95 ≤ TLI ≤ 1.00	0.90 ≤ TLI ≤ 0.95	0.89
IFI	0.95 ≤ IFI ≤ 1.00	0.90 ≤ IFI ≤ 0.95	0.90
RMSEA	0.00 ≤ RMSEA ≤ 0.05	0.05 ≤ RMSEA ≤ 0.08	0.08

Source: [80,81,82,83,84,85].

## Data Availability

The data presented in this study are available on request from the corresponding author.
